# ANTI-IDIOTYPE ANTIBODIES IN IMMUNE REGULATION OF ANCA ASSOCIATED VASCULITIS

**DOI:** 10.4103/0019-5154.55637

**Published:** 2009

**Authors:** Vandana D Pradhan, Kanjaksha Ghosh

**Affiliations:** *From the Department of Autoimmune Disorders, Institute of Immunohaematology, Indian Council of Medical Research, 13^th^ Floor, King Edward Memorial Hospital, Parel, Mumbai - 400 012, India.*

**Keywords:** *Anti-idiotype antibodies*, *ANCA-associated vasculitis*, *Anti-myeloperoxidase antibodies*, *Anti-proteinase3 antibodies*

## Abstract

**Background:**

Anti-idiotype antibodies (anti-ids) have a potential role in the immunomodulation of various autoimmune disorders. The immunoregulatory role of anti-idiotypic antibodies in ANCA-associated vasculitis needs to be studied. This study was conducted in clinically and histopathologically diagnosed ANCA-associated vasculitis (AAV) patients.

**Methods:**

Anti-ids were tested in 100 AAV patients of which 80 had anti-MPO and 20 had anti-PR3 antibodies at various stages of disease over a period of 2-3 years. The disease activity was estimated by the Birmingham vasculitis activity score (BVAS). The affinity-purified ANCA F(ab')2 fragments were prepared using three each of anti-MPO and anti-PR3 high titer sera and were used as idiotype coats for anti-idiotype antibody detection by ELISA. Positivity was confirmed by fluid phase inhibition ELISA.

**Results:**

Patients who went into remission showed 53.8% anti-ids to anti-MPO and 52.9% to anti-PR3 with low BVAS values (0-8), whereas in patients with active disease, only 12.5% had anti-ids to anti-MPO and 10% had anti-ids to anti-PR3 with comparatively high BVAS (18-32), while five cases who had relapse (BVAS 18-20) did not have anti-ids to anti-MPO or anti-PR3. An inverse correlation was noted between ANCA and anti-ids (*r* = −0.901).

**Conclusions:**

High prevalence of anti-ids in remission cases and low prevalence in active cases with absence of anti-ids in relapse cases as well as an inverse correlation of ANCA and anti-ids indicate its beneficial effect on the disease process, thus suggesting the dynamic role of anti-idiotype networks in the immunoregulation of AAV.

## Introduction

Anti-idiotype (anti-id) antibodies to idiotypes (id) that are located in the variable region of autoantibodies have been demonstrated in many autoimmune diseases such as systemic lupus erythematosus, myasthenia gravis, and idiopathic thrombocytopenic purpura. It has been shown that they are important in regulating the pathological immune response, where F(ab')2 components from sera of patients in remission can neutralize the action of related pathogenic autoantibodies. Earlier work on this aspect carried out at this Institute has clearly shown that in SLE, there is an inverse correlation between anti-id and anti-dsDNA autoantibody titers and the clinical manifestations of the disease.[1] Patients having anti-ids had a milder form of disease in comparison with the patients not having anti-ids. An immunosuppression due to the development of anti-ids to the ‘16/6 idiotype’ in about 50% of the patients also has been reported in SLE patients.[2] While in ITP cases, two types of idiotype effects were observed.[3] One was an immune suppression of disease due to the development of anti-idiotype, where 70% cases showed high levels of inhibitory anti-id antibodies and patients went into complete remission as compared to less than 5% cases showing a slight immune enhancement, where patients had lower levels of anti-ids. The disease severity was also found to be inversely correlated to the degree of inhibition of anti-id antibodies. Immunosuppressive role of anti-idiotypes in patients receiving tetanous toxoid has also been reported and there was a significant difference seen in anti-id levels in pre and post immunization cases.[4]

Anti-neutrophil cytoplasmic antibodies (ANCA) are present in the sera of patients with medium to small vessel vasculitides and the changes in the levels of ANCA activity in serum is well correlated with the disease activity.[5] The presence of anti-idiotypes to ANCA could be a helpful tool to explain the possible regulatory mechanism in active and remission sera in patients with ANCA-associated ‘pauci-immune’ vasculitides (AAV). ANCA anti-idiotype activity is present in IgG F(ab')2 fragments purified from ANCA negative remission sera, which inhibits the binding activity of ANCA to its autoantigen in a dose dependant manner.[6] Anti-idiotypes are also found in pooled polyspecific immunoglobulin sera (IVIg), which is mainly used for therapeutic purpose in various autoimmune disorders with beneficial effects.[7]

## Materials and Methods

Clinically suspected cases of vasculitis were referred from the Nephrology, Rheumatology, and Medicine units of some major public hospitals in Mumbai for the present study. These cases were diagnosed as per the Chapel Hill Consensus Criteria (CHCC).[8] These cases were histopathologically proven to be crescentic and/or necrotizing glomerulonephritis with few or no immune deposits. The Birmingham vasculitis activity score (BVAS) was used to assess disease severity in these cases.[9] This prospective study was carried out after obtaining the permission of Ethics Committee. A total of 5ml of clotted blood was aseptically collected from patients at various stages of disease by conducting a follow-up study in every 3-4 months over a period of 2-3 years. The separated serum was stored at −20°C.

Treatment regimen was carefully recorded in these patients. These patients were treated with conventional immunosuppression therapy of oral prednisolone 1mg/kg/day for 3 months and gradually tapering the dose over a year. Cyclophosphamide (CYP) was administered either orally or intravenously. Intravenous CYP was administered on a monthly basis at a dose of 0.5 gm/m^2^ and the dose was adjusted upward to 1 gm/ml^2^ on the basis of leukocyte count at two weeks (>4000/mm^3^). The starting dose was 2mg/kg/day, which was continued until the patient had been in complete remission for at least one year. The dose was then reduced by 25 mg in every 2-3 weeks until either the drug was stopped or a dose reached below which the patient relapsed. None of the patients received intravenous immunoglobulin (IVIgG) therapy. The treatment response was defined as remission, treatment resistance, and relapse. Remission patients showed stabilization or improvement in renal function, resolution of hematuria, and resolution of extra renal manifestations of hematuria and resolution of extra renal manifestations of systemic vasculitis. Relapse cases showed occurrence of at least one of the following symptoms, including rapid rise in creatinine, renal biopsy demonstrating active necrosis or crescent formation, hemoptysis, pulmonary hemorrhage or new or expanding nodules without evidence of infection, and new mononeuritis multiplex or necrotizing vasculitis identified by biopsy. None of the patients showed treatment resistance.

ANCA were detected using human neutrophils (PMN) by the IIF technique.[10,11] Briefly, the method is as follows. PMN were used to prepare a cytospin substrate using Hettich Universal 16 A cytocentrifuge and some slides were fixed with 96% ethanol and others with formalin. After reacting with patient's sera at 1 : 20 dilution, the slides were probed using FITC tagged polyvalent anti-human globulin serum and observed under a fluorescent microscope, Nikon Optiphot II, Japan and microphotography was also done using an automated photography system, Nikon AFX IIA, Japan. The IIF patterns, especially the difficult ones, were also confirmed using a confocal laser scanning microscope (LSM-510, Carl Zeiss, Germany). The specificities of the antibodies were also identified by antigen binding ELISAs for anti-Myeloperoxidase (anti-MPO) and anti-Proteinase3 (anti-PR3) using kits from Genesis, UK. A value <3.0 units/ml was negative, 3-5 units/ml were equivocal and >5 units/ml were considered as positive.

Anti-idiotype antibodies (anti-ids) were studied in 100 clinically and histopathologically proven small- to medium- sized vasculitis cases, of which 80 patients had anti-MPO and 20 had anti-PR3 antibodies during the active stage of disease. Anti-ids were detected in these patients by standardizing idiotype binding ELISA after isolation and affinity purification of IgG by selecting a panel of three high titer anti-MPO and three high titre anti-PR3 positive antigen specific sera. IgG was isolated from whole serum by ammonium sulfate salt fractionation method followed by the DEAE cellulose column chromatography. [12] Affinity purification was done using each IgG subclass antibody using columns of monoclonal antibodies bound to cyanogen bromide activated sepharose 4B columns (anti-IgG1, monoclonal SG-16; anti-IgG2, monoclonal SH-21; anti-IgG3, monoclonal HP-6050; anti-IgG4, monoclonal HP-6025; Sigma, USA).

The affinity purified and antigen specific concentrated IgG was further subjected to pepsin digestion for the preparation of F(ab')2 fragments. The purified F(ab')2 fragments were checked on cellulose acetate electrophoresis and ELISA.[13] This panel of six idiotype coats was used in solid phase anti-id detection ELISA.[6] To obtain the cutoff value, serum samples of 100 normal blood donors were used. Sera from patients with hypergammaglobulinaemia due to other autoimmune and infectious diseases were also used for checking cross relativities. The absorbance values above the mean +2SD were considered as positive. To exclude the presence of cross reactive idiotypes (CRI) and anti-isotype antibodies, anti-idiotype test sera were tested for anti-red cell antibodies, anti-HLA antibodies, and RF.[14–16] All anti-idiotype testing sera were tested in serial dilution from 1 : 10 to 1 : 40 to determine optimum dilution required for the reaction and to exclude the false negative results due to prozone phenomenon. All anti-id positive by ELISA were further confirmed by soluble inhibition method.[17]

The changes in the levels of ANCA activity in serum was tested by regularly monitoring these patients by conducting a sequential follow-up study for 24-28 months at every 4-5 months interval. The changes in ANCA levels were also correlated with the disease activity. The presence of anti-idiotype antibodies that may explain the possibly immunoregulatory mechanism was also detected in active and remission sera of these AVV patients. The additional experiments providing an existence for the ability of the serum obtained from a patient in remission phase that is able to inhibit the binding activity of ANCA to specific target antigen in the serum during acute phase were also performed to confirm the findings of anti-idiotye specificities to anti-MPO and anti-PR3 individually.

## Results

Totally, 100 ANCA positive untreated patients were investigated for anti-id testing, which included 22 patients of RLV (BVAS 8-12), 20 cases of MPA (BVAS 17-30), 20 cases of WG (BVAS 20-30), 20 cases of ICGN (BVAS 15-20), and 18 cases of Class IV LN (SLEDAI 10-30). At the active stage of disease, all these patients were ANCA positive and did not show presence of anti-ids to either MPO-ANCA or PR3-ANCA.

All these patients received the treatment over a period of time and on subsequent follow-ups, 95% patients went into remission and 5% patients showed disease relapse, and none of the patients were found to be disease resistant. Among the remission cases, 82 (86.3%) patients showed presence of anti-ids, of which 69 patients (84.1%) had MPO-anti-ids and remaining 13 patients (15.9%) had PR3- anti-ids [Table 1]. It was observed that 51% patients in remission that had anti-ids tested negative for ANCA, whereas 11% patients in remission had anti-id antibodies also had ANCA levels at low titers (1: 40-1: 80). The incidence of anti-ids was more in remission sera as compared to acute sera and an inverse correlation with ANCA titers and anti-ids was noted (*r* = −0.901). During active stage of disease 18/80 (22.5%) had MPO-anti-ids and only 2/20 WG patients had PR3-anti-ids. During remission, 42/78 patients (53.8%) had MPO-anti-ids and 9/17 (52.9%) had PR3 anti-ids where ANCA was found to be absent in all cases. A small group of 9/78 patients (11.5%) had MPO anti-ids and 2/17 (11.8%) had PR3 anti-ids with low levels of ANCA in them during remission [Table 1].

**Table 1:** ANCA and anti-idiotype antibodies in ANCA-associated vasculitis

Disease status	Anti-myeloperoxidase (*n* = 80)				Anti-proteinase 3 (*n* = 20)			
		
	Number tested	BVAS	ANCA positives (%)	Anti-idiotype positives (%)	Number tested	BVAS	ANCA positives (%)	Anti-idiotype positives (%)
Acute	80	8-30	80	18 (22.5)	20	10-30	20	2(10)
Remission	78	0-8	9(11.5)	42 (53.8)	17	0-8	2(11.8)	9 (52.9)
Relapse	2	18-20	2	0	3	20-25	3	0

* = Clinically highly suspected vasculitis cases but ANCA negative; ** = On follow up after 3–6 months; 8% cases were ANCA positives

In five patients that included two cases of MPA and three cases of WG, ANCA levels persisted throughout the follow-up period and these patients did not have MPO anti-ids or PR3 anti-ids, respectively. Another five patients that showed relapse, which included two cases of MPA and three cases of WG, a rise in ANCA levels were noted just prior to relapse. None of these patients had MPO anti-ids or PR3 anti-ids. The BVAS was found to be elevated up to 18-20 in cases of MPA and 20-25 in WG cases, which had relapse. As shown in Table 2, 80 cases of anti-MPO related vasculitides were tested at the acute stage of disease. BVAS ranged from 8-30 among these patients. All these patients had antibodies to MPO, with strength of 18-60 units/ml. Among these, 18 patients (22.5%) were found to have anti-idiotype antibodies to anti-MPO antibodies. These patients showed milder disease. When these patients (78/80) were in complete remission, their BVAS was 0-8 and only nine patients (11.5%) persisted anti-MPO ANCA with titers ranging from 10-35 units/ml, whereas 42/78 patients (53.8%) developed anti-idiotype antibodies to anti-MPO antibodies [Table 2]. Among this group, two patients had a clinical relapse with a BVAS of 18-20, they showed elevated antibodies to MPO with levels of 30-40 units/ml and anti-idiotype antibodies were found to be absent in them.

**Table 2:** Follow up of anti-MPO positive patients during acute, remission and relapse stages (*n* = 80)

Disease	Number tested	Acute (80)			Remission (78)			Relapse (2)		
				
		BVAS	Anti-MPO positives	Anti-id positives	BVAS	Anti-MPO positives	Anti-id positives	BVAS	Anti-MPO positives	Anti-id positives
RLV	22	8-12	22	6	0-8	4	12	0	0	0
MPA	20	17-30	20	4	0-10	2	10	18-20	2	0
ICGN	20	15-20	20	5	0-8	1	8	0	0	0
Class IV	18	10-30	18	3	0-10	2	12	0	0	0
LN		(SLEDAI)			(SLEDAI)					
Total (%)	80	-	80 (100)	18 (22.5)	-	9(11.5)	42 (53.8)	-	2 (100)	0

RLV = Renal-limited vasculitis, MPA = Microscopic polyangiitis; ICGN = Idiopathic crescentic glomerulonephritis; LN = Lupus nephritis

## Discussion

Various reports have suggested the immunomodulatory role of anti-idiotypes in autoimmune disorders.[19–21] In the present study, some significant points, such as the high incidence of anti-ids to antibodies directed to anti-MPO and PR3, along with low BVAS, the low incidence of anti-ids in patients with active disease having a high BVAS and absence of anti-ids in relapse cases having high BVAS along with an inverse correlation of ANCA with anti-ids antibodies, are emerged.

The presence of anti-idiotypic antibodies to ANCA in patients with SVV has suggested the possible immunoregulatory role of idiotypic regulation. In patients with acute disease, 22.5% had anti-idiotype antibodies to anti-MPO antibodies and 10% had anti-idiotype antibodies to anti-PR3 antibodies. Patients in remission had elevated levels of anti-idiotype antibodies, where 53.8% patients had anti-idiotypes to anti-MPO antibodies and 52.9% had anti-idiotypes to anti-PR3 antibodies. Pall *et al*. 1994 have mentioned that the F(ab')2 fragments of the anti-idiotype antibody inhibit ANCA binding to MPO and further that F(ab')2 fragments of anti-idiotype antibodies bind to F(ab')2 fragments of anti-MPO thus showing the possible immunoregulatory role of idiotypic regulation in the remission sera.[14] ANCA anti-idiotype antibody activities are mainly measured by the inhibition of ANCA binding to their target antigens, and therefore it was important to test anti-idiotype antibodies in ANCA negative sera, mainly in remission cases.

It has been reported that ANCA anti-idiotype antibodies, predominantly of IgG isotype, show spontaneous regulation of ANCA by anti-idiotype antibodies in AAV.[19] Western blot confirmations of anti-idiotypes in this study showed that anti-idiotype antibodies from one patient can interact with the 90-kD isolated F(ab')2–IgG ANCA (id) fragment from the sera of another patient of the same specificity, indicating that ANCA idiotype antibodies are ‘public’, sharing the common epitopes. However, there was no cross reactivity between MPO and PR3 anti-idiotype antibodies. An inverse correlation was noted between ANCA's and anti-idiotype antibodies (*r* = −0.901) in untreated SVV patients with acute disease, where the BVAS was between 8 and 30, and also in remission patients, where BVAS was comparatively low (0-8), a high incidence of 53.8% patients had anti-idiotype antibodies to anti-MPO and 52.9% had anti-idiotype antibodies to anti-PR3 antibodies. RLV patients showing clinical relapse did not show the presence of anti-idiotype antibodies to either anti-MPO or to anti-PR3 antibodies. Follow-up studies of these patients showed that 8% had later developed anti-MPO antibodies and 30.4% had anti-idiotype antibodies to anti-MPO antibodies.

Strunz *et al.* had reported a 50% incidence of antibodies to 5/7 id, which is an anti-PR3 idiotype and *in vitro* suppression of PR3–ANCA antigen binding activity by 5/7 anti-idiotype antibodies were detected in 57.8% patients who were positive for 5/7 idiotype, and they reported that 5/7 anti-id is a promising tool for the development of a specific immunotherapy in WG patients.[20] Jayne *et al*. had reported that 53% patients in remission showed anti-idiotype antibodies to anti-MPO antibodies and there was an inverse correlation between them and reported that remission is not just a passive state due to the absence of disease, but it also depends on the activity of anti-idiotype regulatory mechanisms.[6] While comparing the characteristics of vasculitis and nonvasculitic conditions, Locke *et al*. observed 9/G4 idiotype on anti-MPO antibodies in 60% patients with active vasculitis and 20% of disease control patients, which may indicate the common origin of anti-MPO antibodies in different individuals.[21]

The recent treatment modalities of AAV includes the use of IVIg, which is known to contain anti-idiotype antibodies, and its ability to produce long lasting remission is found to be associated with fall in ANCA levels.[14] IVIg has a potential to influence the pathogenetic process in patients with SVV at different stages of disease and its influence on the idiotypic regulation of ANCA may explain the observed clinical responses and point to possible targets for novel immuno–modulation and for more specific immunotherapy in the near future.[6,7] The induction of anti-idiotype antibodies in experimental animal models to demonstrate systemic vasculitis have also suggested the possible role of anti-idiotype antibody network theory in immunoregulation.[22,23] These observations point to the dynamic interactions that may be taking place between autoantibodies and the complimentary anti-idiotype antibodies in ANCA associated vasculitis (AAV) and also suggests that perturbation of the idiotypic network may represent important fundamental aspect in the regulation of ANCA activity. This can further emphasize and focus on the analogy that may be drawn between therapeutic suppression of ANCA with IVIgG, which can act as immune modulators that may lead to the spontaneous regulation of ANCA activity by anti-idiotypes in AAV and also proposes the possibility of the development of an anti-idiotype immuno suppressive vaccine against these disorders.

## Conclusions

The high prevalence of anti-ids in remission cases and low prevalence in active cases with absence of anti-ids in relapse cases as well as the inverse correlation of ANCA and anti-ids indicate its beneficial effect on the disease process, which in turn suggest the dynamic role of anti-idiotype networks in the immunoregulation of AAV.
